# Generation of Infectious Mimivirus Virions Through Inoculation of Viral DNA Within *Acanthamoeba castellanii* Shows Involvement of Five Proteins, Essentially Uncharacterized

**DOI:** 10.3389/fmicb.2021.677847

**Published:** 2021-07-09

**Authors:** Dehia Sahmi-Bounsiar, Jean-Pierre Baudoin, Sihem Hannat, Philippe Decloquement, Eric Chabrieres, Sarah Aherfi, Bernard La Scola

**Affiliations:** ^1^IHU Méditerranée Infection, Marseille, France; ^2^Aix-Marseille Université, Institut de Recherche pour le Développement (IRD), Assistance Publique- Hôpitaux de Marseille (AP-HM), MEPHI, Marseille, France

**Keywords:** L442, single-cell transfection, microinjection, *Acanthamoeba castellanii*, ApMV, L724, L829, R387

## Abstract

One of the most curious findings associated with the discovery of *Acanthamoeba polyphaga* mimivirus (APMV) was the presence of many proteins and RNAs within the virion. Although some hypotheses on their role in Acanthamoeba infection have been put forward, none have been validated. In this study, we directly transfected mimivirus DNA with or without additional proteinase K treatment to extracted DNA into *Acanthamoeba castellanii.* In this way, it was possible to generate infectious APMV virions, but only without extra proteinase K treatment of extracted DNA. The virus genomes before and after transfection were identical. We searched for the remaining DNA-associated proteins that were digested by proteinase K and could visualize at least five putative proteins. Matrix-assisted laser desorption/ionization time-of-flight and liquid chromatography–mass spectrometry comparison with protein databases allowed the identification of four hypothetical proteins—L442, L724, L829, and R387—and putative GMC-type oxidoreductase R135. We believe that L442 plays a major role in this protein–DNA interaction. In the future, expression in vectors and then diffraction of X-rays by protein crystals could help reveal the exact structure of this protein and its precise role.

## Introduction

*Acanthamoeba castellanii* (Byers, 1979) is a small, complex, and free-living amoeba that can also live as a parasite within a host tissue. In some cases, it is associated with human diseases (Król-turmińska and Olender, 2017). *Acanthamoeba* has been a useful model in various biological studies (Weisman and Korn, 1967; Read and Kabana, 1980; Fouque et al., 2012; La Scola, 2014; Sahmi-bounsiar et al., 2019; Hasni et al., 2019), notably for its membrane capacity to engulf molecules and/or microorganisms whether naturally by phagocytosis of bacteria and viruses (Weisman and Korn, 1967; La Scola et al., 2001; Araú et al., 2016; Raoult and Boyer, 2010) or artificially *via* chemical (Peng et al., 2005; Mougari et al., 2019) or mechanical (Sinard and Pollard, 1989) transfection processes for antibodies, genomic DNA, plasmids, or fluorochrome delivery. Transfection methods have been used for decades (Eisenstark, 1965; Kim and Eberwine, 2010), and among physical transfections, microinjection is a technique that enables the integration of cells or large molecules on a microscopic scale in cells, whether adherent or in suspension culture (Wang et al., 2008; Dean and Gasiorowski, 2010). It has revolutionized the medical field by making *in vitro* fertilization possible (Lopata et al., 1980). In biology, microinjection was one of the first transfection tools used for the study of several cellular processes (Zhang and Yu, 2008). The microinjection of *A. castellanii* has only been carried out once, in 1989, with the aim of studying the motility of this amoeba (Sinard and Pollard, 1989). Due to its complexity, the need for special equipment (Dean et al.) and skilled experimenters, the use of microinjection regressed with the advent of chemical transfection.

With the improvement of the coculture process on amoeba (Yaacoub et al., 2016), our laboratory has greatly contributed to the discovery and improvement of the isolation of giant viruses of amoeba (Rolland et al., 2019) since the discovery of *Acanthamoeba polyphaga* mimivirus (APMV) in 2003 (La Scola et al., 2003). One of the most curious findings associated with the discovery of this virus was the presence of many proteins and RNA within the virion (Suzan-Monti et al., 2007). The involvement of these RNA and proteins has been suggested to be associated with the early stages of infection but has never been fully investigated. The aim of this study was to explore this hypothesis by directly transfecting APMV DNA into *A. castellanii.* Using microinjection, we were able to transfect *A. castellanii* amoeba with mimivirus extracted DNA and generate infectious APMV virions. We revealed the need for DNA-mediated APMV generation of at least four uncharacterized proteins—L442, L724, L829, and R387—and putative GMC-type oxidoreductase R135.

## Materials and Methods

### Cell Preparation

We used *A. castellanii* (ATCC 30010) as a cellular support in peptone–yeast extract–glucose (PYG) medium at a concentration of 5 × 10^5^ cells/ml cultured at 28°C in 75-cm^2^ cell culture flasks, as previously described (Yaacoub et al., 2016). After 48 h of incubation, the flask was gently tapped to detach adherent cells, which were centrifuged for 10 min at 500 × *g* to remove all amoebae debris. The cell pellet was then resuspended and washed twice in starvation medium (Yaacoub et al., 2016). A suspension containing 2 ml of amoebae at 10^3^ cells/ml was then plated into a cell imaging dish (Ibidi glass-bottomed 35-mm petri dish; Germany) with low confluence, allowing for good observation and manipulation control.

### DNA Extraction and Proteinase K Treatment

For mimivirus production, 10 150-cm^2^ flasks containing 10 ml of *A. castellanii* at 5 × 10^5^ cells/ml in 30 ml of PYG were inoculated with 5 ml of *A. polyphaga* mimivirus at a multiplicity of infection of 10. The cocultures were incubated at 30°C and checked daily by inverted optical microscopy to observe cytopathic effects. After the complete lysis of the amoeba cells, the virus supernatant was collected from the cultures and then filtered through 0.8-μm-pore filters to eliminate debris. The supernatant was centrifuged at 14,000 × *g* for 45 min. The supernatant was removed by aspiration, and the pellet was then resuspended in 1 ml of phosphate-buffered saline. The virus was then purified by ultracentrifugation at 14,000 × *g* for 45 min across a 25% sucrose layer, and the viral pellet was resuspended with 1 ml of phosphate-buffered saline and stored at −80°C. Viral DNA was then extracted from 200 μl of the purified virus (10^8^ particles/ml) using the EZ1 advanced XL and using EZ1 DNA Tissue Kit (Qiagen, Hilden, Germany) according to the manufacturer’s instructions. Extracted DNA was doubly filtrated through a 0.22-μm-pore filter and quantified with NanoDrop^TM^ 2000 at approximately 150 ng/ μl. The extracted APMV DNA concentration was diluted to 10 ng/ml for microinjection. To remove the remaining proteins, proteinase K (Thermo Fisher Scientific, United States) treatment was performed by adding to 200 μl of APMV DNA, 200 μl of Tampon G2, and 10 μl of proteinase K. The digestion process takes place at 56°C for 2 h. This second digestion was thereafter referred to as proteinase K extracted DNA pre-treatment.

### Microinjection Components and Procedure

**FIGURE 1:**
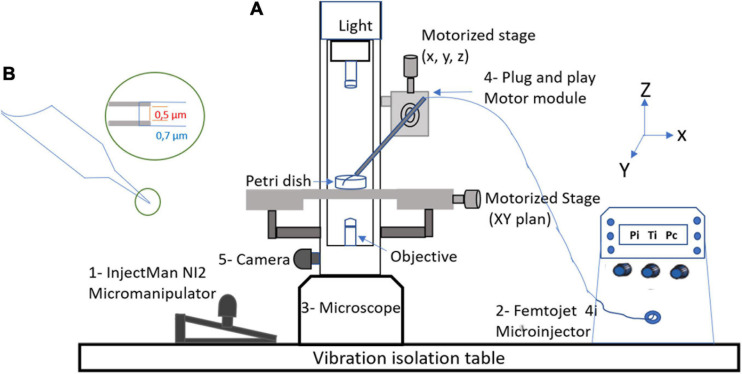
Micromanipulation equipment. **(A)** Components of workstation: 1—InjectMan NI2 micromanipulator which allows for the positioning of the micropipette (Eppendorf Equipment, France), 2—FemtoJet 4i microinjector (Eppendorf, France), 3—Eclipse TE 2000S Inverted microscope (Nikon, France), 4—plug and play motor modules, and 5—DFC 425C camera (Leica, Germany). **(B)** Femtotips with an inner diameter of 0.5 μm.

The workstation (Figure 1) essentially comprises an injectMan NI2 micromanipulator (Eppendorf Equipment, France), which allows the micropipette to be positioned, as well as a femtoJet 4i microinjector (Eppendorf, France), micropipettes Femtotips II (0.5 μm inner diameter and 0.7 μm outer diameter) (Eppendorf, France), an eclipse TE 2000S inverted microscope (Nikon, France), and a DFC 425C camera (Leica, Germany). A computer module was used to observe manipulations and take pictures (Nikon, France). For microinjection, the femtoJet 4i system was used at an injection pressure (Pi) of 75 hectopascals (hPa), a compensation (holding) pressure (Pc) of 10 hPa, and 0.2 s for the time injection (Ti). The microinjection solution was composed of 1 μl of a red fluorescent dye (Dextran Rhodamine B, 70,000 molecular weight, neutral, Invitrogen) and 9 μl of APMV DNA extract at a concentration of 10 ng/μl before injection amoebas were placed into the cell imaging dish (Eppendorf) with 2 ml of starvation medium. Using a microloader, the microinjection needle was filled with 2 μl of the injection solution and then mounted onto an Eppendorf micropipette holder attached to an eclipse TE 2000S inverted microscope (Nikon, France) with an epifluorescence system. The cells were injected by maintaining a constant low flow rate out of the needle tip. The needle was inserted into the amoeba at a shallow angle of −45°, kept in the cell enough to inject about 5–10% of the cell volume, and then removed. Usually, the microinjected volume is on the order of a femtoliter or picoliter. The microinjection volume is determined by the injection parameters (Pi and Ti), the type of femtotips (its opening and shape), and the viscosity of the microinjected solution. Only indirect methods can determine the approximate volume as performed in this study (Keith et al., 1983).

Retention of the fluorescent label was indicative of successful cell microinjection. During each session of microinjection, a negative control consisting of amoeba that were not microinjected was performed by adding 9 μl of APMV DNA extract and 1 μl of red fluorescent dye to fresh amoeba in 2 ml of starvation medium. Afterward, the successfully microinjected amoebas were monitored microscopically for the assessment of the presence of APMV virions released into the medium. The APMV virions could be found after a period of between 1 and 3 weeks, during which the culture medium was changed regularly. Once the presence of virions was noted, cells were scraped from the dish and subcultured along with the resulting culture supernatant in a new petri dish containing a monolayer of fresh amoeba.

### Flow Cytometry Detection

Flow cytometry based on side scatter and DNA content was used. After cytopathic effect and lysis detection, a supernatant was centrifuged at 700 × *g* for 10 min to discard large debris. The supernatant was stained using SYBR green dye (SYBR green I nucleic acid gel stain; Molecular Probes, Life Technologies, Inc., Carlsbad, CA, United States) at a dilution of 1:100 and heated to 80°C for 3 min. Data were collected on a BD LSR Fortessa (BD Biosciences) cytometer and compared with those for previously known gated viruses by using FlowJo software.

### Sample Preparation and Image Acquisition for Scanning Electron Microscopy

The samples were centrifuged at 14,000 × *g* for 10 min, and the supernatant was suspended in 2.5% of glutaraldehyde fixative solution. We then directly placed the sample onto microscopy slides for observation. We used the Hitachi TM4000 scanning electron microscope (SEM) (Hitachi, Japan) for image acquisition.

### Comparative Genomic Analysis

Genomic DNA mimivirus (pre-microinjection and post-microinjection, respectively) was quantified using a Qubit assay with the high sensitivity kit (Life Technologies, Carlsbad, CA, United States) to 0.2 ng/μl. The genomic DNA was then sequenced on MiSeq Technology (Illumina Inc., San Diego, CA, United States) with the paired end strategy and was barcoded in order to be mixed, respectively, with 28 other genomic projects prepared with the Nextera XT DNA sample prep kit (Illumina). Dilution was performed to reach 1 ng of each genome as input to prepare the paired end library. The “tagmentation” step fragmented and tagged the DNA. The limited-cycle PCR amplification (12 cycles) then completed the tag adapters and introduced dual-index barcodes. After purification on AMPure XP beads (Beckman Coulter Inc., Fullerton, CA, United States), the libraries were then normalized on specific beads according to the Nextera XT protocol (Illumina). Normalized libraries were pooled into a single library for sequencing on the MiSeq. The pooled single-strand library was loaded onto the reagent cartridge and then onto the instrument along with the flow cell. Automated cluster generation and paired end sequencing with dual index reads were performed in a single 39-h run in 2 × 250 bp. Total information of 12.47 Gb was obtained from a 642-K/mm^2^ cluster density, with a cluster passing quality control filters of 95.17%. Within this run, the index representation for mimivirus pre-microinjection was determined to be 3.05%, and it was also 3.66% for mimivirus post-microinjection. The 11,864,976 paired end reads were filtered according to the read quality. To compare the genetic contents between both isolates (before and after microinjection), all annotations were performed using the Prokka annotation pipeline (Seemann, 2014) and then used in the Roary pan-genome pipeline using default parameters (Page et al., 2015). The resulting core genome alignments were used to study SNPs using SNP-Sites (Page et al., 2016). These results were double-checked by an in-house script for detecting SNPs.

### Identification of DNA-Associated Proteins

We used both silver and Coomassie blue staining originally developed to detect proteins separated by SDS-PAGE (Switzer, 1979; Merril et al., 1981; Heukeshoven and Dernick, 1985).

Five protein bands were excised manually from Coomassie Blue staining gels. After several successive washes with acetonitrile and water, in-gel digestion with proteomics-grade trypsin (Agilent Technologies) was done overnight at room temperature. The peptides obtained from protein digestion were extracted with acetonitrile.

Peptides were identified as a first step using MALDI-TOF-MS (matrix-assisted laser desorption/ionization time-of-flight mass spectrometry) on a Bruker Autoflex Speed spectrometer (Bruker Daltonics) and as a second step using a nanoAcquity UPLC system connected to a Synapt G2Si Q-TOF spectrometer (Waters).

For the MALDI-TOF analyses, 1 μl peptide mixture was cocrystallized onto the Anchorchip MALDI-TOF target plate with an equal amount of matrix solution (0.3 mg/ml of α-cyano-4-hydroxycinnamic acid in acetone/ethanol, 1:2 v/v, acidified with TFA, 0.1% final). The mass spectrometer was calibrated externally using bovine serum albumin tryptic peptides. The peptide mass fingerprints were used to identify the proteins.

For the liquid chromatography–mass spectrometry (LC-MS) analyses, peptides were pooled and were eluted onto a trapping column (nanoAcquity UPLC 2G-V/M Trap 5μm Symmetry C18 180 μm × 20 mm, Waters) for concentration and desalting at 10 μl/min of 99.9% water, 0.1% formic acid, 0.1% acetonitrile, and 0.1% formic acid. The peptides were eluted on a C18 100 μm × 100 mm column (nanoAcquity UPLC 1.7 μm BEH C18, Waters) and separated using a 100-min gradient (300 nl/min, 5–40% acetonitrile, and 0.1% formic acid). Data-dependent MS/MS monitoring was performed in positive mode. GFP lock mass correction was applied to spectra. Raw MS data was processed using PEAKS Studio 6.0 software. Swissprot online protein sequences were used for protein identification. Proteins presenting one or more peptides were considered as identified.

A tertiary structure prediction was used for all the uncharacterized proteins—L442, L724, L829, and R387—using Phyre2 tool (Kelley et al., 2015).

## Results

### Production of APMV Virions After Microinjection of APMV DNA in Amoeba

**FIGURE 2:**
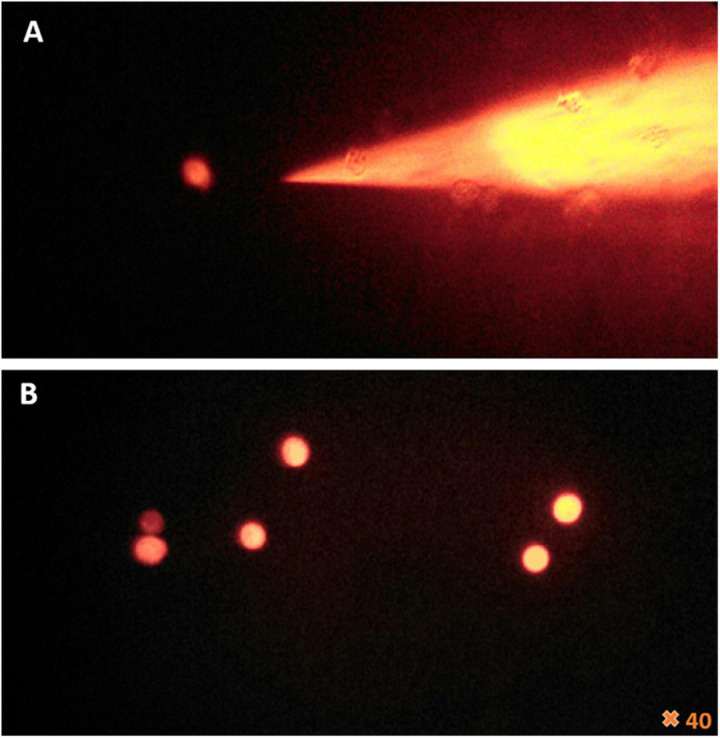
Fluorescence microscopy of the femtotips and the cell’s reaction to microinjection. **(A)** Illustration of femtotips removed from microinjected amoeba. **(B)** Observation of amoeba stained in red by rhodamine-dextran and the effect of the microinjection which induces the loss of amoeba adherence and their rounding.

**FIGURE 3:**
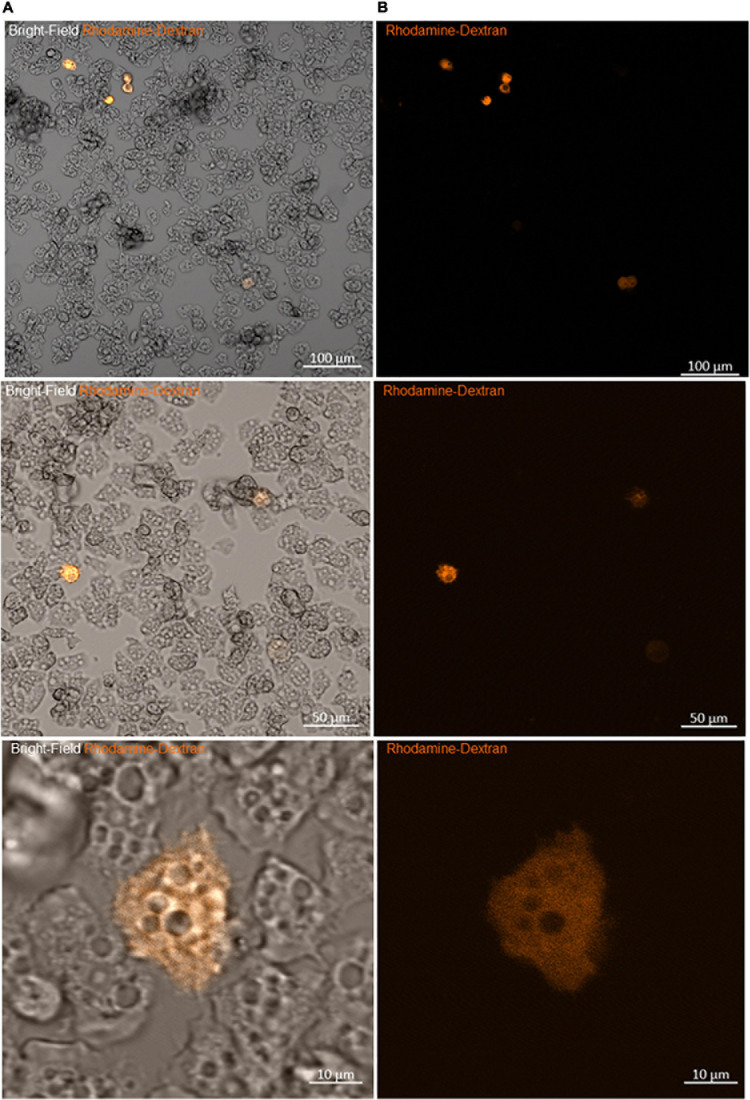
Viability of amoeba microinjected with rhodamine-dextran dye. **(A)** Bright-field images showing the maintenance of the trophozoite state of different amoebae 2 h after microinjection. **(B)** Fluorescence microscopy images of the same frame show the homogeneity of the red dye into the cytoplasm, without any captation by other structures, sign of the successful entry of DNA and the success of the microinjection.

**FIGURE 4:**
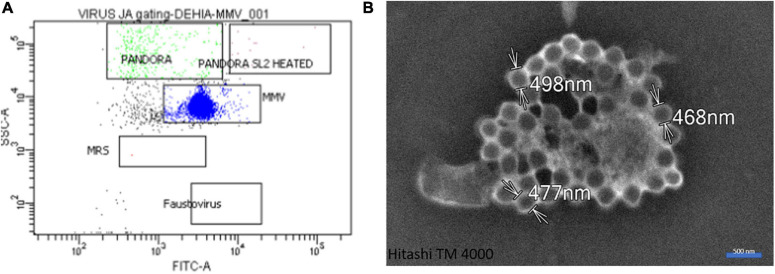
Gating and microscopic control. **(A)** Flow cytometry dot plot showing the single viral profile in SSC (side scatter) versus FITC (SYBR green DNA contents) of mimivirus. **(B)** Scanning electron microscopy with Hitachi TM4000 of a culture supernatant showing mimivirus particles.

Our microinjection methodology was successful in a quarter of the 200 sessions performed. Successful experiments made it possible to achieve between one and eight microinjected amoebae, as checked by fluorescent-dextran loading of the cells (Figures 2, 4). Of these 50 successful experiments, six led to amoeba monolayer lysis associated with the production of mimivirus virions. Despite the shock reaction with cell rounding and detachment from the substrate that was initially observed optically (Figure 2), the successfully microinjected cells recognized by the fluorescent cytoplasm recovered their normal morphology within 1–2 h after microinjection (Figure 3). A confirmation of cell morphology, motility, and viability was performed 24 h post-microinjection (Supplementary Video 1). The viability, as checked by observing cell morphology and motility, was comparable to non-microinjected cells. None of the negative controls led to amoeba lysis. Of the six microinjections leading to the production of viral particles, a cytopathic effect consisting of slight lysis or rounding of a fraction of the amoeba population was observed, starting from 5 to 7 days post-microinjection. A subculture of the supernatant and cells of these dishes on fresh amoeba was then carried out and followed by daily optical observation. Amoeba lysis at the second passage was observed after 2–4 days, with the production of viral particles as detected first with optical microscopy and then confirmed by SEM and flow cytometry. SEM (Hitachi TM4000) showed viral particles presenting the same morphological characteristics as APMV and with mean maximal diameters between 460 and 500 nm (Figure 4B). Flow cytometry dot plot also confirmed the production of mimivirus, showing in SSC (side scatter) versus FITC (SYBR green DNA contents) a single viral population corresponding to the size/structure profile of mimivirus virions and quantified at 10^8^ particles/ml (Figure 4A). Genome sequencing was performed on purified mimivirus solution and on viral particles produced by amoeba after microinjection. Sequence analysis confirmed that both analyzed genomes were that of *Acanthamoeba polyphaga* mimivirus (GenBank access number AY653733) and revealed 100% similarity between the two viral isolates (Supplementary Figure 1).

### APMV Virion Production by Microinjected Amoeba Is Blocked by Proteinase K Treatment of APMV DNA and Involves Certain Proteins

To check if some proteins associated with APMV DNA are necessary for viral production, a step of pre-treatment with proteinase K was added to the DNA after its extraction in order to eliminate any residual protein. In parallel, 50 microinjection sessions with pre-treatment with proteinase K and 50 sessions without pre-treatment were carried out. Out of 50 sessions without proteinase K treatment, we obtained 12 successful experiments, each one making possible to achieve between one and five microinjected amoebae. Two of these led to viral production. In contrast, no microinjection with proteinase K pre-treated APMV DNA led to amoeba monolayer lysis associated with APMV particle production.

**FIGURE 5:**
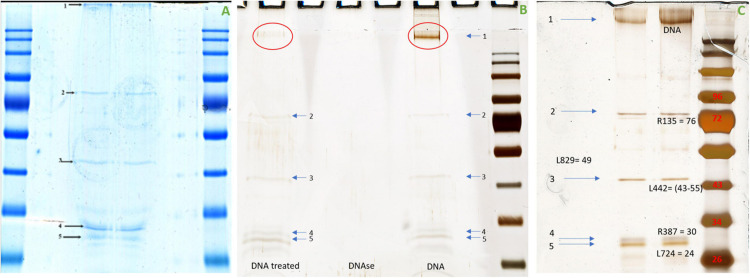
Migration of the APMV DNA extract into polyacrylamide gel electrophoresis. **(A)** Coomassie blue staining (InstantBlue Protein Stain) of polyacrylamide gel electrophoresis showed five different bands possibly corresponding to proteins. **(B)** Silver staining of polyacrylamide gel electrophoresis showed the disappearance of band no. 1 when the APMV DNA is treated with DNase and the persistence of the band nos. 2, 3, 4, and 5. **(C)** Silver staining of polyacrylamide gel electrophoresis showed five bands corresponding, respectively, to DNA, R135 putative GMC-type oxidoreductase (molecular weight = 76,947 Da), cleaved sequence of the hypothetical protein L442 (molecular weight ≃ 48,000 Da), hypothetical protein R387 (molecular weight = 30,067 Da), and hypothetical protein L724 (molecular weight = 24,033 Da). No visible band corresponds to hypothetical protein L829 (molecular weight = 49,229 Da). The protein marker used was Color Prestained Protein Standard, Broad Range (10–250 kDa).

To better understand the nature of the proteinase K digested material from APMV DNA extract, protein analysis was carried out using SDS-PAGE. This analysis revealed five constant putative protein bands (Figures 5A,C). In-gel digestion and MALDI-TOF-MS showed the presence in a single band (band no. 3) of the cleaved sequence of an uncharacterized protein, L442 with a size between 43 and 55,223 kDa (Figure 5C). Band no. 1 corresponds to DNA, as indicated by its removal after DNase treatment, while the other bands persist (Figure 5B). The intensities of band nos. 2, 3, 4, and 5 were too low to be analyzed with MALDI-TOF. This result was confirmed by those obtained by LC-MS which give as first hit one L442 (139,334 Da) with 11% coverage for 12 identified peptides. Putative GMC-type oxidoreductase R135 (76,947 Da) was also found with 16% coverage for 10 identified peptides. Other mimivirus proteins could also be identified with one peptide for uncharacterized protein R387 (30,067 Da) and two for uncharacterized proteins L724 (24,033 Da) and L829 (49,226 Da) (Supplementary Figure 2).

**FIGURE 6:**
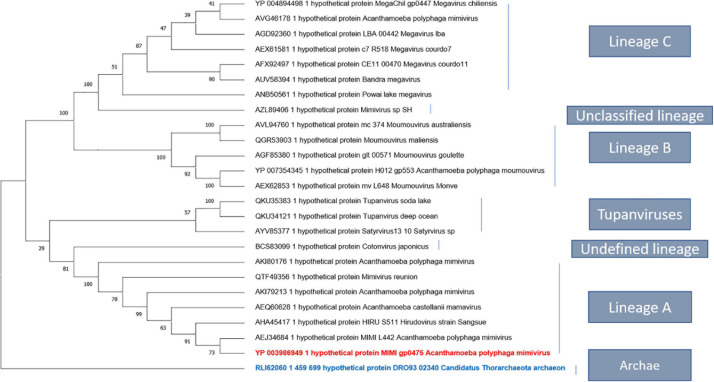
Phylogenetic tree based on the amino acid sequences of mimivirus uncharacterized protein L442. The analysis was performed using maximum likelihood method (ML). The amino acid sequences were aligned using Muscle, and the tree was built using FastTree. Bootstraps below 50 were discarded. There were a total of 1,358 positions in the final dataset. Evolutionary analyses were conducted in MEGA X.

Homologs of uncharacterized protein L422 were present in lineage A, B, and C of mimiviruses and in tupanviruses. A more distant homolog that we detected by delta Blast is also present in an archaeon, in agreement with an analysis of the relationship between Nucleo-Cytoplasmic Large DNA Viruses (NCLDVs) and cell domains concluding on the emergence of NCLDVs before the last eukaryotic common ancestor (Guglielmini et al., 2019). A phylogenetic analysis of L422 was performed either without outgroup (Supplementary Figure 5) or using the archaeal as an outgroup (Figure 6). The results suggest that L422 was already present in the common ancestor of Mimiviridae and Tupanvirus.

The proteins L829 and R387 also have homologs in the three lineages of mimiviruses and in tupanviruses, and phylogenetic analysis suggests that they were already present in the common ancestor of these viruses (Supplementary Figures 5–7).

The putative GMC-type oxidoreductase R135 and the uncharacterized protein L724 have only homologs in the three lineages of mimivirus, and the phylogenetic analysis suggests that it was already present in the common ancestor of the A, B, and C lineages (Supplementary Figures 3, 4).

Using phyre2 tool, a tertiary structure prediction applied first to the entire sequence of L442 then to the cleaved sequence, showed both similarity with human ATP-dependant DNA helicase q5 (first hit; Confidence: 47.2% Identity 32%) and (first hit; Confidence: 48% Identity 32%), respectively. These scores were too low to predict any putative function, but both organisms are also phylogenetically distant. This result suggests that this protein sequence may be involved in DNA metabolism (Supplementary Figure 8).

Using the same tool, a tertiary structure applied to the sequence of L724, L829, and R387 showed a similarity related, respectively, to the field of transcription regulation (first hit; confidence: 65.2%, identity: 42%), hydrolase (first hit; confidence: 91.2%, identity 32%), and cysteine zipper (first hit; confidence: 94.9%, identity: 20%). The scores stay low to predict any putative function (Supplementary Figure 8).

## Discussion

**FIGURE 7:**
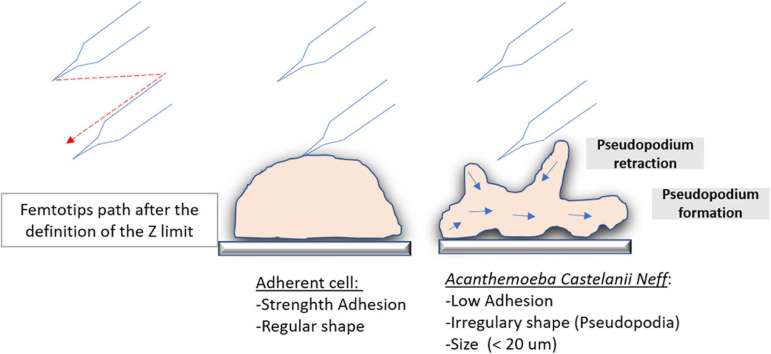
Microinjection difference on highly adherent cells and on amoeba. Schematic illustration of the trajectory comparison of the femtotips on a highly adherent cell and on an amoeba that is a less adherent cell during the initiation of the microinjection.

Our microinjection methodology for the inoculation of APMV DNA extract into *A. castellanii* has proven to be an efficient, albeit tricky, method for inducing infectious APMV virion production. We also showed that at least one protein associated with APMV DNA is required for APMV production after DNA microinjection. Expertise and the repetition of multiple experiments was necessary for an efficient microinjection setup, leading, in this case, to the efficient production of viral particles after microinjection of APMV DNA. Virions produced by microinjected amoeba were microscopically identified as mimiviruses, checked by flow cytometry, and confirmed by genome sequencing, which showed that initial mimivirus and microinjection-produced mimivirus were identical. The major drawback of this methodology was a low success rate that could be explained by various parameters. The low diameter of *A. castellanii* cells (<20 μm) and their constant morphological changes with the formation and retraction of pseudopodia (Figure 7) led to diverse individual cell heights and difficulties in setting Z-limits for microinjection. The amoebae also exhibit a very weak adhesion on surfaces, potentially causing detachment by capillary. We also noticed that amoebas were able to maintain microinjected samples into vacuoles and that cell membranes could be perforated, resulting in physical damage of the cells with capillary clogging. It should be noted that concentrated APMV DNA extract generated cytotoxic effects on amoeba cells, leading to the use of a diluted APMV DNA extract for further microinjection. Low concentrations of APMV DNA extracts may result in a lower performance of the microinjection. The various manipulations of DNA (extraction, double filtration, mixing the extract with the dye, loading it in femtotips, and then injecting femtotips) raise questions as to its integrity, but previous work has shown a genomic reduction of 16% for mimivirus after several passages (Boyer et al., 2011), suggesting that all genomic integrity is accessory to viral production. These technical complications cannot all be resolved, giving rise to poor performance. One of the strategies used by amoeba and avoided by microinjection is the external signaling of phagocytosis (Silva et al., 2016). This work reported that putative quorum-sensing molecules secreted by trophozoites infected with giant viruses induce the transition of neighboring cells to the cyst-resistant phase, giving them protection against giant viruses. Indeed the microinjection of an amoeba avoids the emission of the signal encystment factors, rendering the amoeba susceptible to infection. In addition to the infection of only one or some cells (less than nine cells) with the microinjection system, the absence of phagocytosis and external signaling could be the result of the slow multiplication of the virus that is followed first by the cytopathic effect of the amoeba and then by amoeba lysis with viral production.

For the first time, we have shown that the microinjection of viral DNA into its host cell leads to the production of APMV virions. This production of APMV virions was prevented by the pre-treatment of APMV DNA with proteinase K before microinjection. Protein analyses with MALDI-TOF and LC-MS were carried out, which demonstrated five interesting proteins—uncharacterized proteins L422, L724, L829, and R387 and putative GMC-type oxidoreductase R135 seem particularly interesting. Uncharacterized protein L422 was probably associated with APMV DNA and was therefore necessary for viral production after the microinjection of APMV DNA. The presence in uncharacterized protein L442 of glycosylated bonds between aa365 and aa410 (NSS, NST, and NNS) (generated with the Bruker biotools software) raises questions about other possible proteins. It would be interesting to understand whether this protein, found in a cleaved state, is in its native state or why it is in this state. However, this work on protein analysis needs further investigation. The similarity of the three-dimensional structure of this protein to a human ATP-dependent DNA helicase suggests that this protein could be involved in the access of the amoeba replication machinery to the dsDNA extracted by APMV. The most widely reported and best described DNA-related proteins in giant viruses are homologs of core histones, which are found in the viral particles of Marseilleviridae (Erives, 2017), *Acanthamoeba castellanii* medusavirus (Yoshikawa et al., 2019), and Clandestinovirus (unpublished data). In our work, these histones were not detected on viral DNA.

Previous studies have already identified certain proteins found in our work. On the one hand, Boyer et al. (2011) identified two glycosylated proteins (L829 and R135) representing an antigenic part of Mimivirus fibrils. Concerning these same fibrils, Araújo et al. (2015) have described an original mechanism corresponding to their strong adhesion which is mediated by glycans, specifically mannose and N-acetylglucosamine (a monomer of chitin and peptidoglycan), allowing their attachment to different organisms, especially to amoebae and virophages. Without this adhesion capacity of the fibrils, mimiviruses will not interact with virophages and amoebae (Boyer et al., 2011). In addition, mimivirus protein R135, associated to fibrils, was found in the protein panel of the virophage Sputnik (La Scola et al., 2008).

On the other hand, Bekliz et al. (2018) demonstrated that silencing of the R458 gene, encoding the R458 protein predicted for the initiation of translation, induces a deregulation of the expression of 32 proteins. Among the five proteins identified in this work, four of them—uncharacterized proteins L442, L724, and L829 and putative GMC oxidoreductase R135—are included in this set of deregulated proteins. Indeed these protein deregulations are generally associated with viral particle structure, transcription machinery, oxidative pathways, protein/lipid modifications, and DNA topology and repair. L442 with unknown function was found in both cases, with nine spots downregulated against four spots upregulated. L724, with unknown function, and R135, involved in oxidative pathway, were found in the upregulated spots. L829, with unknown function, was found in the downregulated spots (Bekliz et al., 2018).

## Conclusion and Perspective

Inoculation of APMV DNA extract into *A. castellanii* by microinjection has proven to be an efficient, albeit tricky, method of inducing infectious APMV virion production. Giant virus virions not only encompass RNA, dsDNA, and proteins but also DNA-associated proteins, whose role is mandatory for infecting cells after DNA microinjection. Uncharacterized proteins L422, L724, L829, and R387 and putative GMC-type oxidoreductase R135 may be involved in APMV dsDNA availability to process through the amoeba replication machinery, and it would be interesting to express them in order to study its structure. This innovative methodology with APMV DNA extract microinjection into amoeba may be more broadly applied to other giant viruses or even non-giant classical DNA viruses and thus represents a powerful tool in the field of virology. This microinjection method should make it possible to further analyze the relationships between giant viruses, amoebae, and especially virophages. Due to the limited size of their genome, indeed virophages are more easily genetically modified. In fact, it becomes potentially possible by using this microinjection technique to inject the DNA of the susceptible giant virus and that of the modified virophage at one time. It thus becomes possible to study the effect of each virophage gene (by KO, for example) or to test the effect of these modifications on the capacity of the MIMIVIRE system to destroy the virophage. Microinjected amoebae can further be individually cloned by combining single-cell microaspirations (Sahmi-bounsiar et al., 2019).

## Data Availability Statement

The original contributions presented in the study are included in the article/Supplementary Material, further inquiries can be directed to the corresponding author/s.

## Author Contributions

DS-B performed the microinjection experiments and wrote the manuscript. J-PB performed the development of microinjection experiments and wrote the manuscript. PD performed the proteomics experiments and analyzed the data. EC and SA wrote the manuscript. BL designed and supervised the study and wrote the manuscript. All authors read the final version of the manuscript.

## Conflict of Interest

The authors declare that the research was conducted in the absence of any commercial or financial relationships that could be construed as a potential conflict of interest.

## Supplementary Material

Supplementary Video 1 Amoeba viability and motility 24 h after microinjection.
