# Supplementary material for: Generation of Infectious Mimivirus Virions Through Inoculation of Viral DNA Within Acanthamoeba castellanii Shows Involvement of Five Proteins, Essentially Uncharacterized
Source: Front Microbiol. 2021 Jul 9;12:677847. doi: 10.3389/fmicb.2021.677847 (PMC8299487; doi:10.3389/fmicb.2021.677847)
Supplement: Supplementary file 2 [file Data_Sheet_1.PDF]

frontiers

Mimivirus 22 vs Mimivirus 26

	Genome length	G+C content	No. of Scaffolds
Mimivirus 22	1,229,162 bp	28%	15
Mimivirus 26	1,230,623 bp	28.1%	15
	**Comparative Genomic Analysis**		
Pan-genome	1,053 genes		
Core-genes	1,043 genes (99%)		
Unique genes	10 genes (1%)		
MMV22 (5/10 unique genes)	NADH-quinone oxidoreductase subunit M<br>hypothetical protein (x2)<br>IS607 family transposase ISvMimi_1 (x2)		
MMV26 (5/10 unique genes)	Cytochrome c oxidase subunit 1<br>hypothetical protein (x4)		
Core-genes-based-alignment SNP	(0) nucleotide		

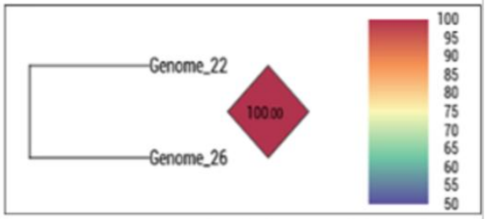

Figure 1: Genome similarity matrix based on OrthoAni analysis

Supplementary Figure S1. Sequence similarity analyzed between pre-microinjection Mimivirus (22) and post-microinjection Mimivirus (26)

Oxidation (M) (+15.99)

```
1    MRGNNLRGGV GEYKINDVLG TRFQNFMDLV DQQKLTPFLV RNLDASLASA TGSPYGLDDL HPDFAEYVMA VSAALRQIES
81   PELTKVQVTG KYGNALESIV NDLFSGTTSG LIAQPTQPFG FNQLPFHNNH PNIKHLIPGF HLFPYTSAVY PTVNQNNNAL
161  INAILYLYHY ISLLDLDSGV DARNIVSFLK KDNLVFNRYV NDIVTTMTNN NFFSQSLGFP TGQPTDEIVR DSVLQGLTGV
241  AYQIVNRLRN VQASNQTFAN LGAVAGQDIS AKDAFAKFVS SVATPGYPGV NADKLFDAFS KITGGFYDQG PDVVNIADVD
321  NDQAKQYRTN GLLNPVYILS PSIAKTVNAD DYDKLNQAGG KRNSSMNNST QNNNSSRSNN SARNNNSVWN NNNSAWKNNN
401  SAWNDNSSWK NNNRFGQAGG ITFGDIGAAP AGSVLSLPFL YGPALPDGSH KLITEDEQGD QNDAKLVDID SVKEIEDLTD
481  PNNITGIIPE IVDIGNDPNY NYARAQLVTL LYQLIVNEAT FDQASGLPNL DNEIRNAAQN YNKIVVRLRA IPTSNFGSSM
561  ADLRDSFLSE FVNTSYRQFQ VEKQTGNLIT GQQTIANKGS NFANPDIKSF YDNTLVNNAD FYKTYFNLVK LGPNGVVVDA
641  DVKDITEAKG KSDAELQNYR LNVRKNTGYT RFTGAQVGGL LGDIVFIDRI PAFPQDGSIR NVWLTRAIAL TPVTLNAYNV
721  EALRRIAREV YNSPVGQSTV PVYGQPVDLT LIAQSAARMN FPISNVAFRD TFNNLLQNAL NQAATGTAVT PGFIEQEDKL
                                                                              889
801  VEHLLRVSSR WERDGNTFIF KDLSGNPVQT DPADNCLLID TSTRECLSVL TQCIADPGTK LSDTCARLME FNFKVNPPLN
881  LLKDEISKMN PGVAFEILRK FGFGSYLAED KDDSGSVIRR YKVQSVGSWI RELMGESARC APGQAPVVNQ GPCRTISLRE
961  ELGQANADKI LNMAKDSAPF LRYLEVLVHW VNANPQVLNP EETKDQSTLC PTSYPKVNDS FNTYSYLNPY KDVVYRLRNT
1041 TCDLERLKCS IMGNYLGSGS RKIFTDLATI PHDTNMPFTR IGFTSTVPLL NKVPMFGGDG GIYNLQNQLN NLNNPVGYNM
1121 FHQIYKDLLN TMGNIGDSRC IRLSSNTQAR CEDKLESFKN AEIELNKCLN RLIERNKIYQ ATRGRIDLNR VPPENVAAVL
1201 EKHSNLLNMN SAYNKKAVNL IDIFQTIAKA IINKVEEGAP KQTVERPLTM GFHNPSYNF
```

L422

```
1    MKNKECCKCY NPCEKICVNY STTDVAFERP NPCKPIPCKP TPIPCDPCHN TKDNLTGDIV IIGAGAAGSL LAHYLARFSN
81   MKIILLEAGH SHFNDPVVTD PMGFFGKYNP PNENISMSQN PSYSWQGAQE PNTGAYGNRP IIAHGMGFGG STMINRLNLV
161  VGGRTVFDND WPVGWKYDDV KNYFRRVLVD INPVRDNTKA SITSVALDAL RIIAEQQIAS GEPVDFLLNK ATGNVPNVEK
241  TTPDAVPLNL NDYEGVNSVV AFSSFYMGVN QLSDGNYIRK YAGNTYLNRN YVDENGRGIG KFSGLRVVSD AVVDRIIFKG
321  NRAVGVNYID REGIMHYVKV NKEVVVTSGA FYTPTILQRS GIGDFTYLSS IGVKNLVYNN PLVGTGLKNH YSPVTITRVH
401  GEPSEVSRFL SNMAANPTNM GFKGLAELGF HRLDPNKPAN ANTVTYRKYQ LMMTAGVGIP AEQQYLSGLS PSSNNLFTLI
481  ADDIRFAPEG YIKIGTPNIP RDVPKIFFNT FVTYTPTSAP ADQQWPIAQK TLAPLISALL GYDIIYQTLM SMNQTARDSG
561  FQVSLEMVYP LNDLIYKLHN GLATYGANWW HYFVPTLVGD DTPAGREFAD TLSKLSYYPR VGAHLDSHQG CSCSIGRTVD
641  SNLKVIGTQN VRVADLSAAA FPPGGNTWAT ASMIGARAVD LILGFPYLRD LPVNDVPILN VN
```

R135

```
1    MKICFEKNFE CCNPCQPKCC PPPCPPKCCP PPCPPKCESI CIDKCPNTCD PCCPPLVDDC LAKKLECLWR QCFCDARLIP
81   EFGVPCQSDG VAVITHTLGR GLCNLKINGL KSQSILANNS FYSVEVSGCK WLNLYEIQLP DVPGKNGCKS SGEIYTEALV
161  KLGISVEGDG YRWKGSQPYC LNIHSKAIGM HPIEFSKKQI AAIKAVLDYF FCDNKCCC
```

L724

```
1    MSNRFDSKPK CRCVAKIDDN YENNCQSKYI SKCEIPRNIC QRKNIDFFYD FRLKYSDADF DYVFGNDGVV TQNFTGLTVN
81   SVPFTQTVPI GNEHPKWLKF YKDAFPLYND REVIFETEMS GVQVIDGNSI PEKMKPRIRN VDDDLRLASG ALNVIDPNTW
161  MVFDFFVTNT AIYAFYERLP FGKTSSTPSN TTSQFGNKSF HDKFTHNGSI HNGSIHNGSI HNGSHCNPNP DVPTDLGNYA
241  AFSNAIWVAR RSADDPLSQF SKLAIGIHKG KGLVTWYIDD IPVFTWDRIG YRMHDEYRMV DHGGIEGIVS PDSMRLGFGT
321  FSLFDMNLPN DYDRGYVDPV VVLPDGPHRE IARSALIQLD FAANYRETFP DPYTGLERPL ADPAITFAYT LGETPDDNRA
401  IKLFGQGAII KLKYLRVYTR SPNAKPEFSR VNH
```

L829

```
1    MDKKTWVYII IAIIIILLLV WYFRNHMSDQ KGVNVNNQTY NMLQQQISSL NQQILFLKQQ ISNLHVPAPT STVNSLRQTV
81   SDINQQVSTI NNQISSLNPY LPRNQQLELA SVLSIFNRNA LDLNNISRSV INRDINYFNA GQHGSQVPQN SHTVQNPNVA
161  DNELNVLQQK VDNLNGVVSN IRQHLAQFGS GIPESFRDEA EKAASYLNDR IDDINKNLPN LVQRLNPNQR NNLNRILSEL
241  NNDLSSLKNS LGSAVRNRIN SVNIH
```

R387

Supplementary Figure S2. Protein coverage of Uncharcterised protein L442 (12 petptides), L724 (2 petptides), L829 (2 petptides), R387 (1 petptide) and Putative GMC-type oxidoreductase R135 (10 petptides). The probably cleaved sequence of uncharcterized protein L442 was selected between red brakets ((Sequence including all peptides identified by MALDI-TOF. (Molecular weight ≃ 48000 Da, calculated using ProtParm tool))

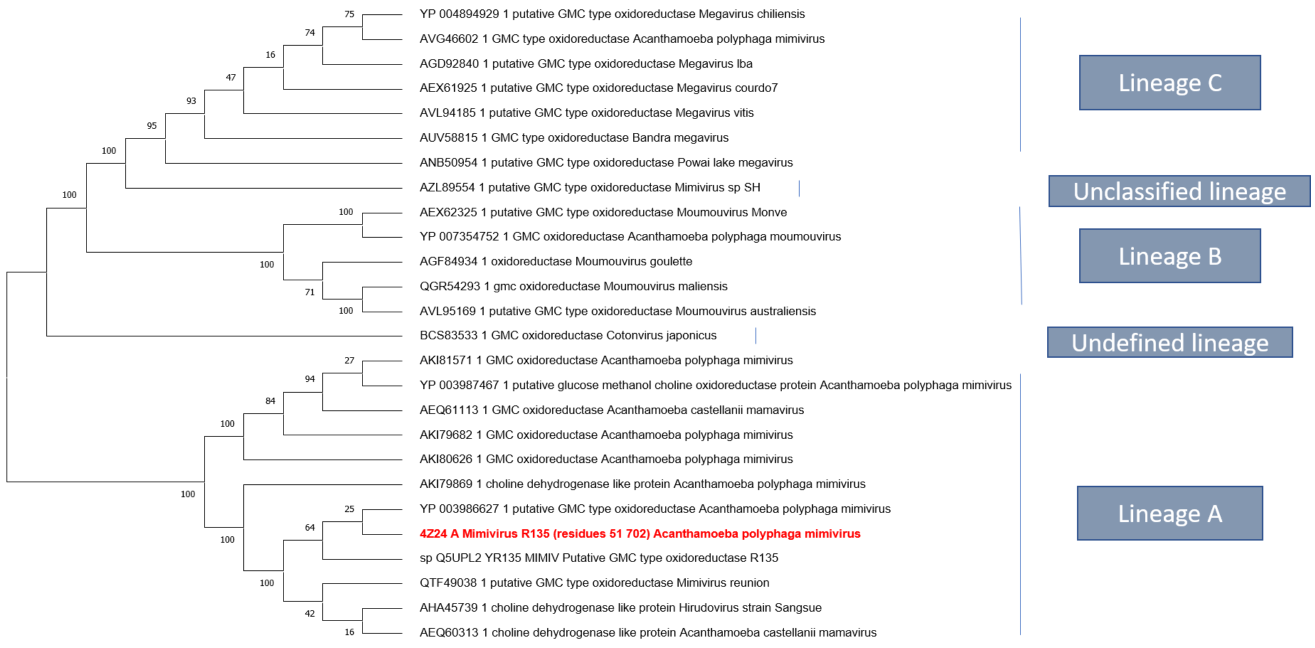

Supplementary Figure S3. Phylogenetic tree based on the amino acid sequences of putative GMC-type oxidoreductase R135. The analysis was performed using Maximum Likelihood method (ML). The amino acid sequences were aligned using Muscle and the tree was built using FastTree. This analysis involved 26 amino acid sequences. There was a total of 649 positions in the final dataset. Evolutionary analyses were conducted in MEGA X

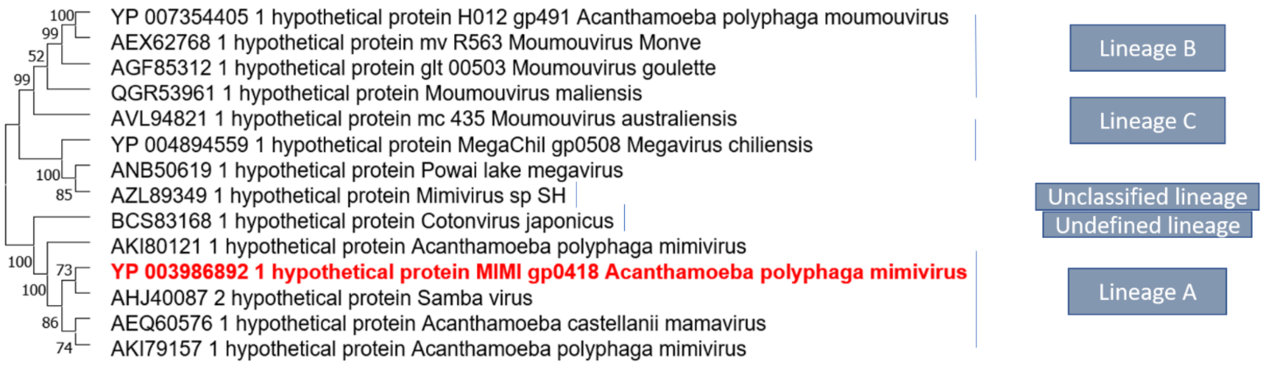

Supplementary Figure S4. Phylogenetic tree based on the amino acid sequences of uncharacterised protein L724. The analysis was performed using Maximum Likelihood method (ML). The amino acid sequences were aligned using Muscle and the tree was built using FastTree. This analysis involved 14 amino acid sequences. There was a total of 206 positions in the final dataset. Evolutionary analyses were conducted in MEGA X

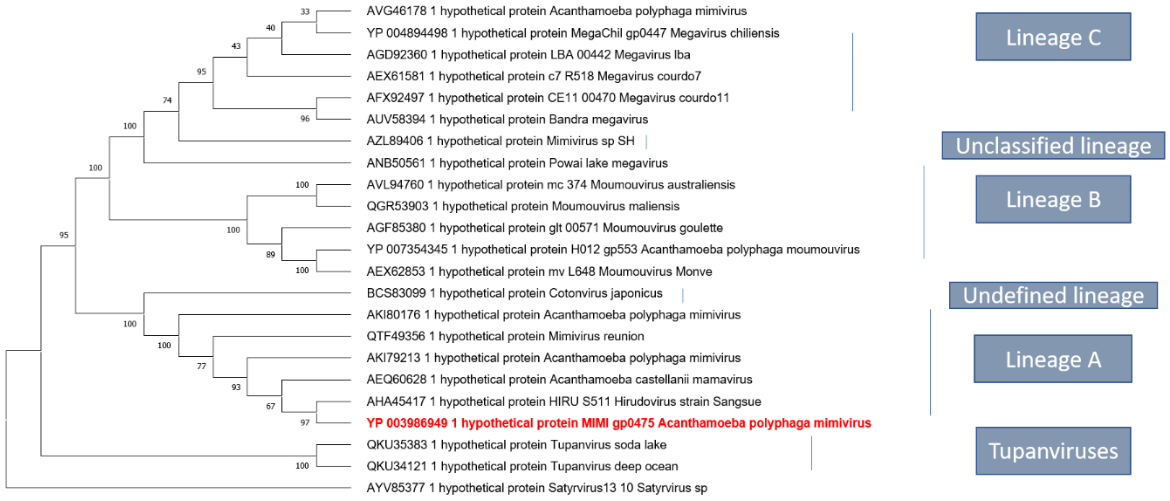

Supplementary Figure S5. Phylogenetic tree based on the amino acid sequences of uncharacterised protein L442. The analysis was performed using Maximum Likelihood method (ML). The amino acid sequences were aligned using Muscle and the tree was built using FastTree. This analysis involved 23 amino acid sequences. There was a total of 1404 positions in the final dataset. Evolutionary analyses were conducted in MEGA X

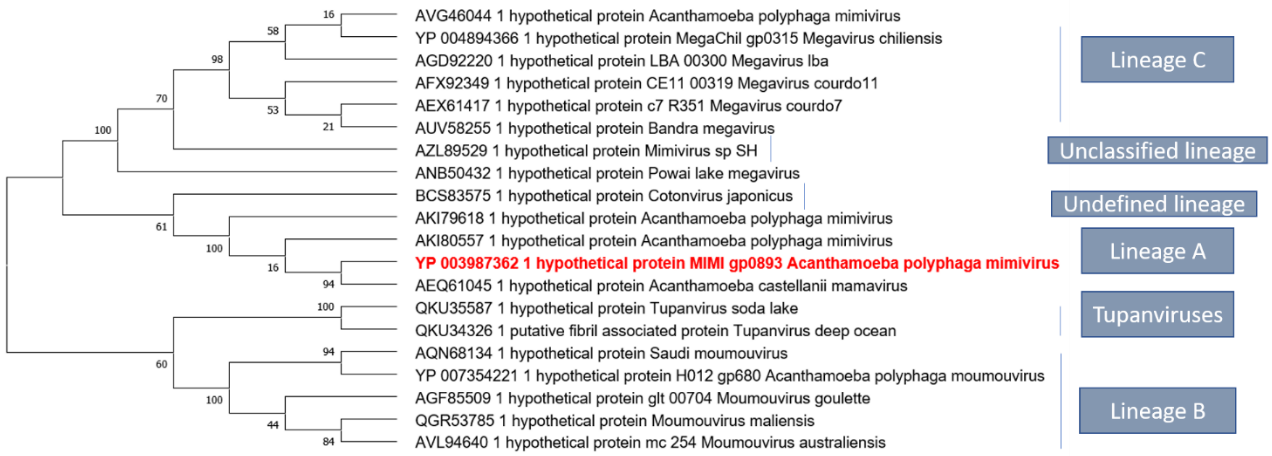

Supplementary Figure S6. Phylogenetic tree based on the amino acid sequences of uncharacterised protein L829. The analysis was performed using Maximum Likelihood method (ML). The amino acid sequences were aligned using Muscle and the tree was built using FastTree. This analysis involved 20 amino acid sequences. There was a total of 233 positions in the final dataset. Evolutionary analyses were conducted in MEGA X

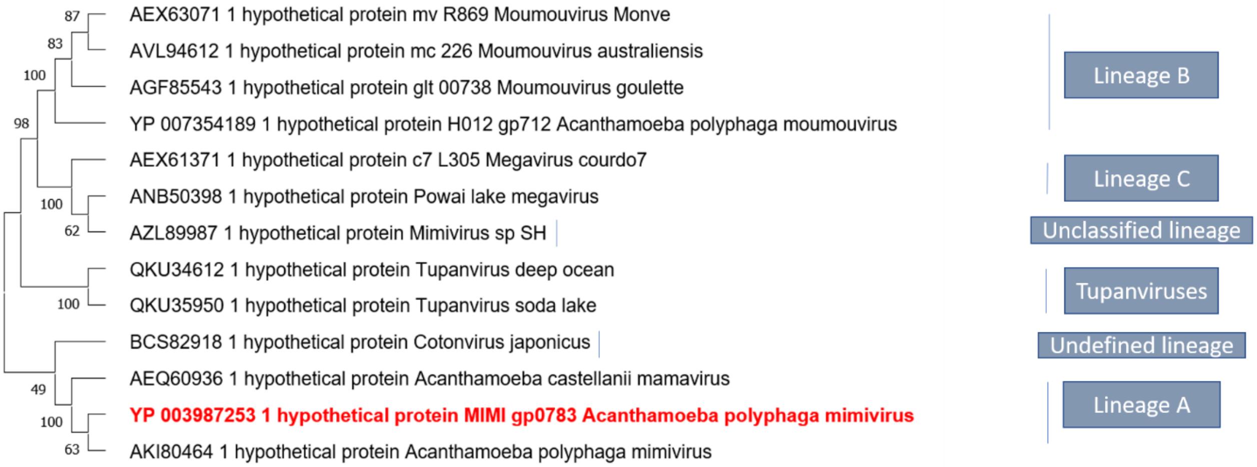

Supplementary Figure S7. Phylogenetic tree based on the amino acid sequences of uncharacterised protein R387. The analysis was performed using Maximum Likelihood method (ML). The amino acid sequences were aligned using Muscle and the tree was built using FastTree. This analysis involved 13 amino acid sequences. There was a total of 158 positions in the final dataset. Evolutionary analyses were conducted in MEGA X

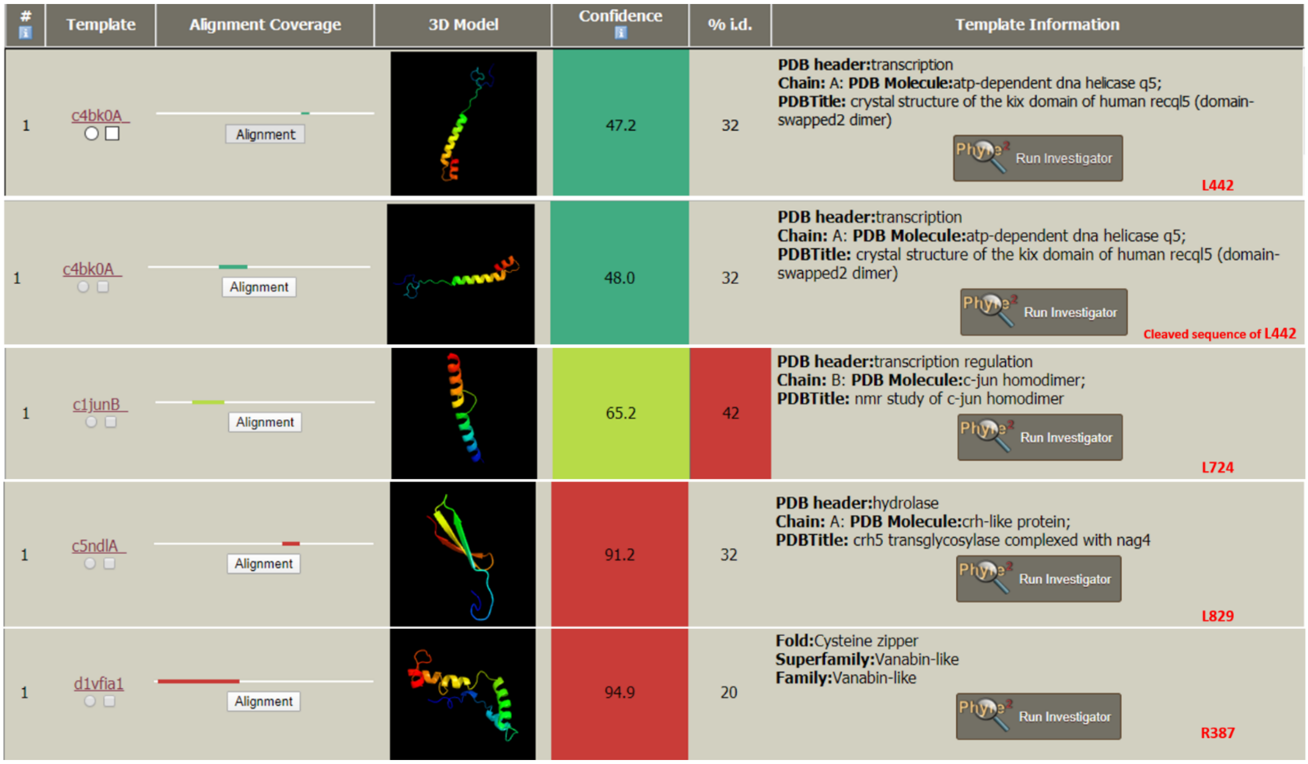

#	Template	Alignment Coverage	3D Model	Confidence	% i.d.	Template Information
1	c4bk0A	Alignment		47.2	32	**PDB header:**transcription **Chain:** A: **PDB Molecule:**atp-dependent dna helicase q5; **PDBTitle:** crystal structure of the kix domain of human recql5 (domain-swapped2 dimer) Run Investigator L442
1	c4bk0A	Alignment		48.0	32	**PDB header:**transcription **Chain:** A: **PDB Molecule:**atp-dependent dna helicase q5; **PDBTitle:** crystal structure of the kix domain of human recql5 (domain-swapped2 dimer) Run Investigator Cleaved sequence of L442
1	c1junB	Alignment		65.2	42	**PDB header:**transcription regulation **Chain:** B: **PDB Molecule:**c-jun homodimer; **PDBTitle:** nmr study of c-jun homodimer Run Investigator L724
1	c5ndlA	Alignment		91.2	32	**PDB header:**hydrolase **Chain:** A: **PDB Molecule:**crh-like protein; **PDBTitle:** crh5 transglycosylase complexed with nag4 Run Investigator L829
1	d1vfia1	Alignment		94.9	20	**Fold:**Cysteine zipper **Superfamily:**Vanabin-like **Family:**Vanabin-like Run Investigator R387

Supplementary Figure S8. Protein prediction with phyre2 for Uncharcterised protein L442 (Entire and cleaved sequence), L724, L829 and R387.
